# Prevalence of alcohol use disorders in individuals with borderline personality disorder: a meta-analysis and meta-regression study

**DOI:** 10.1590/1516-3180.2024.0480.R1.04112025

**Published:** 2026-02-09

**Authors:** Stefani Gonzalez Silva, Maria Olivia Pozzolo Pedro, Joao Mauricio Castaldelli-Maia

**Affiliations:** I Physician, Departamento de Neurociência, Faculdade de Medicina, Centro Universitário FMABC, Santo André (SP), Brazil.; II Physician, Departamento de Psiquiatria, Faculdade de Medicina, Universidade de São Paulo (FMUSP), São Paulo (SP), Brazil.; III Associate Professor; Physician; Departamento de Psiquiatria, Faculdade de Medicina, Universidade de São Paulo (FMUSP), São Paulo (SP), Brazil.

**Keywords:** Alcoholism, Epidemiology, Borderline personality disorder, Alcohol use disorders, Borderline disorder, Borderline, Borderline and alcohol

## Abstract

**BACKGROUND:**

This review examined the prevalence rate of alcohol use disorders (AUDs)–including heavy episodic drinking, heavy drinking, alcohol abuse, and alcohol dependence–among individuals with borderline personality disorder (BPD).

**OBJECTIVES:**

The primary objective of this meta-analysis and meta-regression study was to investigate the prevalence AUDs associated with BPD.

**DESIGN AND SETTING:**

We searched PubMed, Google Scholar, Virtual Health Library (VHL/BVS), SciELO, LILACS, EMBASE, and PsycINFO for studies, reports, or abstracts published without language restrictions.

**METHODS:**

We searched for reports published from database inception through March 2024. This study followed the Preferred Reporting Items for Systematic Reviews and Meta-Analyses (PRISMA) and Meta-analysis Of Observational Studies in Epidemiology guidelines (MOOSE). Based on the extracted data, we performed meta-analyses and meta-regressions.

**RESULTS:**

The final sample included 15 articles with 15,603 individuals aged 18 years or older with BPD. The prevalence of AUDs with BPD was 55.28%, while the prevalence of alcohol dependence (AD) was 44.59%, and alcohol abuse (AA) was 18.84%.

**CONCLUSION:**

Our findings indicate a high prevalence of AUDs among individuals with BPD, underscoring the need for targeted prevention and treatment strategies. Integrated dual-diagnosis approaches addressing both disorders simultaneously are crucial for improving outcomes. This high prevalence has important implications for public health.

## INTRODUCTION

 Alcohol consumption is a major public health concern associated with numerous health problems and a high percentage of mortality^1^ Several factors can influence alcohol consumption, and although the prevalence of alcohol use disorder (AUD) in individuals with borderline personality disorder (BPD) has not been well established, emerging evidence suggests increased susceptibility in this population.^2^

 AUD is defined by compulsive alcohol use, impaired control over consumption, and negative emotional states during withdrawal, and it often becomes chronic and recurrent.^3^ According to the DSM-5, "alcohol use disorder" replaces the DSM-4 categories of alcohol abuse and dependence, and is now classified as mild, moderate, or severe.^4,5^ AUD frequently occurs with psychiatric disorders, including personality disorders, further worsening patient outcomes. 

 BPD is classified in the DSM-5 as a Cluster-B personality disorder and is characterized by pervasive affective instability, impulsivity, interpersonal difficulties, and disturbances in self-image.^6 ,7^ Individuals with BPD often exhibit heightened emotional reactivity and sensitivity to social and interpersonal stressors, contributing to significant psychological distress and functional impairment. 

 This meta-analysis examined the prevalence of AUD among individuals with BPD with the goal of informing interventions aimed at reducing alcohol-related harm. It synthesizes findings from population-based surveys reporting lifetime comorbidity rates of BPD and AUD. 

## METHODOLOGY

### Review guidelines and registration

 This study followed the PRISMA statement for transparent reporting of systematic reviews and meta-analyses^8^ and the MOOSE guidelines for meta-analysis of observational studies in epidemiology.^9^

**Figure 1:**
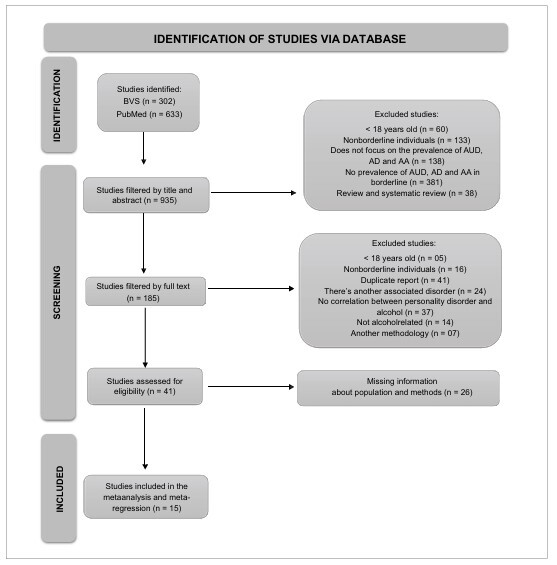
Study’s selection flow chart.

 Both checklists are provided in the supplementary materials (**Figure 1** **and** **2**), detailing where each item is addressed. This study was registered with the Center for Open Science/Open Science Framework ( https://osf.io/6c5np?mode=&revisionId=&view_only=). 

**Figure 2:**
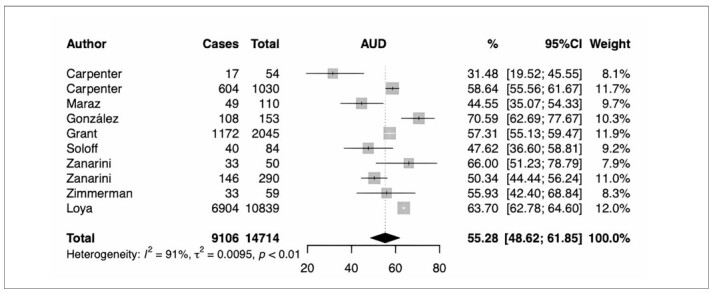
Subgroup analysis of alcohol use disorders.

### Information sources

 Following Cochrane methodology, we searched seven databases–PubMed, EMBASE, Google Scholar, Biblioteca Virtual em Saúde (BVS), SciELO, LILACS, and PsycINFO)–between November 2023 and March 2024 for studies published up to January 15, 2024. No language restrictions were applied. The final search was conducted on March 10, 2024. 

 Medical Subject Headings (MeSH) use included: (alcohol use OR alcoholism OR binge drinking OR alcohol use disorder OR hazardous drinking OR alcohol abuse OR alcohol dependence) AND "(("prevalence"[Mesh]) OR "epidemiology"[Mesh]) AND "borderline disorder"[Mesh]" 

 Health Sciences Descriptors (DeCS) terms were also used: "(epidemiologia) OR (prevalência) AND (alcoolismo) OR (beber em binge) OR (abuso de álcool) OR (alcoolismo) AND (transtorno de personalidade borderline)". In PICO terms, the Population was adults aged ≥ 18 years; the Intervention/Determinant was borderline disorder; the Comparison was without borderline disorder; and the Outcome included AUD, alcohol abuse and alcohol dependence. Books and dissertations were excluded. 

### Selection criteria

 Studies were included if they met the following criteria: (i) crosssectional and longitudinal observational design; (ii) assessment of Alcohol Dependence (AD) Alcohol Abuse (AA), and AUD using validated questionnaire, such as SCID, ICD, MINI, PAI-BOR, PDQ4+, SADS, DIPD-IV, or clinical assessment scales; (iii) participants aged ≥ 18 years; and (iv) no language restrictions. 

 Articles were selected based on the title and abstract, and then reviewed through full-text analysis. All abstracts were independently assessed by two authors, with disagreements resolved by consensus. 

### Data extraction

 Two reviewers independently extracted data, with a third reviewer consulted if needed. Extracted variables included authors, year of publication, total number of participants with a diagnosis of AD, AA, or AUD, total number of participants with BPD, sex, age, study design, country, diagnostic instruments, and diagnostic criteria. 

### Quality assessment

 Methodological quality was assessed using the Joanna Briggs Institute checklist for analytical cross-sectional studies,^10^ and was applied to all studies registered in the current systematic review. The checklist evaluates sample structure, process, size, description of the context, coverage of data analysis, valid and reliable evaluation methods, appropriate statistical analysis, and adequate response rate. Fifteen studies scored ≥ 6 (maximum = 8 points) and were therefore retained (supplementary material, **Table 1**). 

**Table 1:** Descriptive summary of the included studies

**Author (year)**	**Study population**	**Setting**	**Diagnostic criteria**	**Prevalence rates (N)**
Carpenter et al. (2017)^12^	N: 54	COMMUNITY	DSM-IV	AUD 31.48%
	F/M: 4.4			
	USA			
	MEAN AGE: 26.02			
Carpenter et al. (2016)^13^	N: 1030	COMMUNITY	DSM-IV	AUD 58.64%
	USA			
Tadic et al. (2009)^24^	N: 159	CLINICAL	DSM-IV	AD 49.69%
	F/M: 2.2			
	EUROPE			AA 11.95%
	MEAN AGE: 33.45			
Picci et al. (2012)^14^	N: 62	CLINICAL	DSM-IV	AD 83.87%
	F/M: 0.631			
	EUROPE			
Maraz et al. (2016)^14^	N: 110	COMMUNITY	ICD-10/DSM-IV	AUD 44.55%
	EUROPE			
Dulit et al. (1990)^22^	N: 137	CLINICAL	DSM-III	AD 15.33%
	F/M: 4.1			
	USA			AA 33.58%
	MEAN AGE: 29			
Stepp et al. (2005)^42^	N: 356	CLINICAL	DSM-IV	AUD 36%
	F/M: 1.3			
	USA			
	MEAN AGE: 18			
González et al. (2019)^15^	N: 153	COMMUNITY	DSM-IV	AUD 70.59%
	EUROPE			
	MEAN AGE: 37.54			
Grant et al. (2008)^16^	N: 2045	CLINICAL	DSM-IV	AUD 57.31%
	USA			AD 41.56%
				AA 15.7%
Soloff et al. (1994)^17^	N: 84	CLINICAL	DSM-III-R	AUD 47.62%
	F/M: 2.652			
	USA			
	MEAN AGE: 26.9			
Walter et al. (2009)^25^	N: 175	CLINICAL	DSM-IV	AD 34.86%
	F/M: 2.9			
	EUROPE			AA 17.14%
	MEAN AGE: 32.1			
Zanarini et al. (1989)^18^	N: 50	CLINICAL	DSM-III	AUD 66%
	F/M: 1.941			
	USA			
	MEAN AGE: 29.2			
Zanarini et al. (2011)^19^	N: 290	CLINICAL	DSM-III-R	AUD 50.34%
	F/M: 25.363			
	USA			
	MEAN AGE: 27			
Zimmerman et al. (1999)^20^	N: 59	CLINICAL	DSM-IV	AUD 55.93%
	F/M: 1.565			
	USA			
	MEAN AGE: 32.6			
Loya et al. (2024)^21^	N: 10839	COMMUNITY	DSM-V	AUD 63.7%
	F/M: 1.315			
	USA			

*F/M, proportion; USA, United States of America

### Data analysis

 We first determined the prevalence of AD, AA, and AUD among individuals with BPD. Heterogeneity test (Q-test) was used to determine whether the differences between the prevalence estimates in the studies were greater than those predicted by chance. Significant heterogeneity prompted the use of random-effects models. Univariate analyses were performed to assess the relationships between each variable. These included methodological factors, age, sex, and geographical location of the study participants. The combined prevalence of AUD was estimated using a meta-regression approach. Variability in the estimate of AUD prevalence was assessed using a random-effects regression model. A significance level of 5% was used for all the analyses. 

 The prevalence and 95% confidence intervals (CIs) were found for the numbers of AD, AA, and AUD related to BPD. The contribution of each study to each meta-analysis was assessed using sensitivity analysis. R software version 3.5.0 was used to analyze the data. The significance threshold was calculated for p-values below 0.05 (P < 0.05). 

 Statistical regression models have been used in studies where people are considered as the unit of analysis to assess how one or more covariates relate to a dependent variable.^11^ The use of meta-regression instead of the AUD subgroup analysis enabled the inclusion of continuous covariates and only one covariate at a time. Radom effects meta-regression measures the variance between studies in a modified Knapp–Hartung model using restricted maximum likelihood residuals.^12^ Permutation tests were used to correct for multiple testing by calculating the adjusted p-values after analyzing all covariates (sex, age, region, and diagnostic criteria).^12^

## RESULTS

**Figure 1** shows the study selection process. A total of 935 records were screened by title and abstract. Of these, 750 articles were considered for abstract and full-text reading. All abstracts were reviewed by the first author, and some were selected for further review based on the following criteria: (1) articles with BPD individuals, (2) articles focusing on AUD, AA, and AD prevalence, or (3) original articles evaluating AUD, AA, and AD prevalence in samples diagnosed with BPD. In total, 184 articles underwent full-text review. After exclusions–including age < 18 years (n = 5), no BPD diagnosis (n = 16), presence of other associated disorder (n = 24), duplicates (n = 41), no assessment of the BPDalcohol relationship (n = 37), not alcohol-related (n = 14), and methodological incompatibility (n = 7). **Table 2** (supplementary material) presents the main findings of the included studies. 

**Table 2:** Results of the meta-regression models for alcohol use disorders among individuals with borderline personality disorders

**Covariate**	**Coefficients**	**Upper bound**	**Lower bound**	**Std. error**	**P value**
Year	0.001	0.016	−0.013	0.007	0.847
Female	−0.467	0.147	−0.147	0.314	0.136
Age	−0	0	−0	0	0.341
Type	Clinical (reference)				
Community	−0.012	0.387	−0.362	0.191	0.947
Region	Europe (reference)				
U.S.	0.008	0.346	−0.329	0.172	0.959
Criteria	DSM (reference)				
Mixed	0.1	0.445	−0.245	0.176	0.569

 Fifteen unique studies met the inclusion criteria. The final sample comprised 15,603 individuals with BPD, age ≥ 18 years. The studies were classified as clinical (n = 10) and community (n = 5). These data are presented in **Table 1**. 

 The studies were conducted in 6 countries, with the United States contributing to the largest proportion (n = 10). Diagnostic criteria for AUD and BPD varied across studies, mostly commonly DSM-IV (n = 10). Others used DSM-V, DIB, DIPD-IV, DIPD-R, ICD-10, DSM-III, SADS, MCMI-III, PAI-BOR, SCID-I, MINI, AUDIT, or PDQ4+. Six articles were selected based on three criteria (ICD-10, AUDIT, DSM-IV, PAI-BOR, SCID-I, MINI, PDQ4+, DSM-III, DIB, SADS, and DIPD-IV). Three articles were selected based on two different criteria (DSM-IV, MCMI-III, DSM-III, and SCID). 

**Figure 3:**
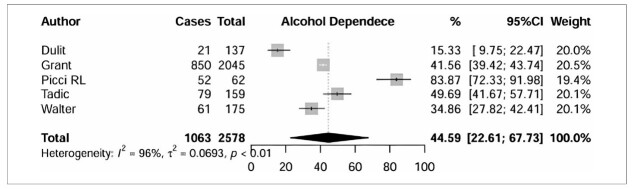
Subgroup analysis of alcohol dependence.

**Figure 4:**
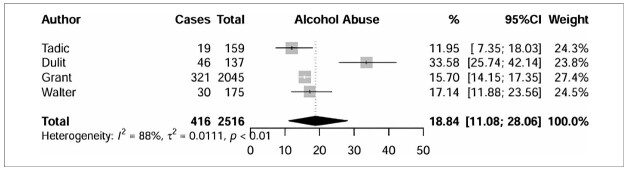
Subgroup analysis of alcohol abuse.

 Figure 2 shows that 55.28% (95% confidence interval [95% CI] = 48.62–61.85%) of the BPD were diagnosed with AUD,^13-22^ 10 studies included the prevalence of AUD. The lowest AUD prevalence was 31.48% (95% CI = 19.52%–45.55%),^13^ while the highest prevalence was 70.59% (95% CI = 62.69%–77.67%).^16^ The pooled prevalence of AD^17,23-26^ in individuals with BPD (**Figure 3**) was 44.59% (95% CI =22.61%–67.73%), and the subgroup analysis investigated five studies involving 1063 individuals. In **Figure 4** four studies investigated AA^17,23 ,25,26^ prevalence among individuals with BPD (n = 2516) and obtained a pooled prevalence of 18.84% (95% CI =11.08%–28.06%). The regression analysis (**Table 2**) revealed no statistically significant variables. 

 Geographic location was significantly associated with the prevalence of AUD, AA, and AD. The prevalence of AUD in North America was 80% (eight studies) and 20% in Europe (two studies). The prevalence rates of AA were 50% in Europe (two studies) and 50% in North America (two studies). The prevalence rates of AD were 60% in Europe (three studies) and 40% in North America (two studies). 

## DISCUSSION

 To the best of our knowledge, no previous systematic review or meta-analysis has investigated the co-occurrence of alcohol dependence (AD), alcohol abuse (AA), and alcohol use disorder (AUD) in individuals with borderline personality disorder (BPD). This meta-analysis sought to synthesize the available evidence to address this gap and provide a comprehensive understanding of the prevalence and combined patterns of AD, AA, and AUD in individuals with BPD. We also explored the possible relationships, clinical implications, and targeted interventions. 

 Our findings indicate that individuals with BPD have a higher risk of AUD relative to the general population. For comparison, data from 2016 estimated AUD prevalence at 8.6% among men (95% CI:8.1%–9.1%) and 1.7% of women.^3,27^ In contrast, our pooled estimates revealed substantially higher prevalence rates among individuals with BPD: 55.28% for AUD, 18.84% for AA, and 44.59% for AD. These results demonstrates a significant burden of comorbid alcohol-related disorders in this population. 

 The rationale for conducting this meta-analysis stems from both the lack of comprehensive investigations on this topic and the profound public health impact of alcohol misuse. Harmful alcohol use accounts for approximately 3 million deaths annually–representing 5.3% of all global mortality,^28^ and is linked to wide range of psychiatric conditions, including personality disorders. AUD trajectories varies considerably: some individuals experience transient episodes, whereas others exhibit patterns of relapse and remission or a persistent and chronic course.^29^ These patterns not only poses health risks but also impose extensive burdens on public health systems, social services, law enforcement, and administrative infrastructures.^30^ Furthermore, AUD frequently coexists with other psychiatric disorders, such as bipolar disorder,^31^ and more than 30% of individuals with AUD present with at least one additional psychiatric diagnosis.^32^

 BPD is frequently underdiagnosed but may be present in up to 6.4% of adults in primary care visits, four times higher than in the general population^7^. It is also associated with numerous medical and psychiatric comorbidities, including obesity, excoriation (skin picking) disorder, and substance use disorders, including alcohol.^33-35^ Studies have indicated that individuals with BPD are more susceptible to developing AUD, largely due to emotional dysregulation, impulsivity, and heightened sensitivity to interpersonal stressors.^1,36,37^

 The high prevalence of AUD among individuals with BPD likely reflects a complex interplay between emotional, cognitive, and genetic factors. Self-damaging impulsivity–a core feature of BPD–has been identified as a strong genetic risk factor for AUD, even more predictive than categorical BPD diagnosis.^38^ Moreover, coping- and conformity-related drinking motives appear to mediate the association between BPD and alcohol-related problems, suggesting that individuals with BPD often use alcohol as a maladaptive strategy for emotion regulation and social belonging.^39^ Emotional dysregulation also plays a key role as BPD individuals show greater mismatches between physiological and subjective emotional responses, which is associated with more frequent alcohol use.^40^ Interestingly, although both BPD and BPD+AUD groups display high levels of impulsivity and maladaptive schema modes, these domains do not differ significantly between groups, indicating shared vulnerability mechanisms regardless of alcohol use.^37^

 In addition, evidence highlights that impulsivity and affective dysregulation contribute not only to AUD comorbidity but also to poorer treatment outcomes. This underscores the need for comprehensive, multimodal interventions that incorporate social network support, psychoeducation, and targeted treatments for both BPD and AUD.^41, 42^ As the clinical importance of empirical data on the co-occurrence of BPD and AUD remain fragmented, our review identified substantial gaps across regions and a lack of large-scale epidemiological studies. 

 Our findings also reveal substantial heterogeneity in reported prevalence across studies. This variability highlights the need for further research to identify underlying mechanisms and contextual factors influencing these differences. Addressing AUD in individuals with BPD represents a pressing clinical priority, as targeted interventions may reduce alcohol-related harm and improve overall treatment outcomes in this high-risk population. 

### Limitations

 This meta-analysis has several limitations. Although the study used broad measures, heterogeneity could not be fully explained by the moderators. Four studies did not stratify participants by sex, instead analyzing as a single population,^13,15-17^ which limited our ability to assess sex-specific patterns. Additionally, data were insufficient to examine all regions; in the lack of studies in Africa, South America, Asia, and Oceania highlights the need for more geographically diverse research. 

 Five studies lacked adequate information on age distribution, restricting age-related analyses.^14,15,17 ,22,24^ One study did not differentiate between AUD, AA and AD among individuals with BPD, reporting them collectively; this study was therefore excluded from the meta-analysis.^43^

 Small sample sizes in some studies may have limited the statistical power needed to detect significant differences. In addition, social stigma associated with reporting alcohol consumption may have contributed to the underreporting of alcohol consumption, especially in specific ethnic groups. The lack of a standard diagnostic method is a limitation of this study. In addition, Google Scholar limits the results of any search to the 1000-most cited papers, potentially omitting relevant but less frequently cited studies. 

## CONCLUSION

 The high prevalence of AUD among individuals with BPD highlights the critical need for early detection and integrated treatment approaches. Individuals with AUD and BPD face increased risks of developing other physical and emotional comorbidities. Therefore, treatment strategies should target both conditions concurrently to mitigate harm and improve clinical outcomes. Future research should explore the interaction between BPD and AUD using diverse methodological approaches, as well as the correlation between AUD and other psychiatric disorders–such as major depressive disorder and substance use disorder–aiming to improve treatment outcomes, reduce harm, and improve public health outcomes. 

## Data Availability

The data that support the findings of this study, including supplementary tables and figures, are available at the Center for Open Science (OSF) repository at  https://osf.io/5mb6f/overview.
