# ERRATUM: Prevalence of alcohol use disorders in individuals with borderline personality disorder: a meta-analysis and meta-regression study

**DOI:** 10.1590/1516-3180.2024.0480.R1.04112025erratum

**Published:** 2026-04-06

In the manuscript "Prevalence of alcohol use disorders in individuals with borderline personality disorder: a meta-analysis and meta-regression study", DOI: https://doi.org/10.1590/1516-3180.2024.0480.R1.04112025, published in the Sao Paulo Med J. 2026;144(1):e2024480.

**Where it read:**

"Sources of funding: None."

**It should read:**

"Sources of funding: This study was supported by the Fundação de Amparo à Pesquisa do Estado de São Paulo (FAPESP), grant number 2023/08459-4."

